# Prognostic value of occult lymph node metastases in patients with completely resected esophageal squamous cell carcinoma

**DOI:** 10.1038/s41598-020-79073-9

**Published:** 2020-12-15

**Authors:** Shao-bin Chen, Di-tian Liu, Shu-jie Huang, Hong-rui Weng, Geng Wang, Hua Li, Yu-ping Chen

**Affiliations:** 1 Department of Thoracic Surgery, Cancer Hospital of Shantou University Medical College, 7 Raoping Road, Shantou, 515000 Guangdong China; 2 Department of Clinical Laboratory, Shantou Hospital Traditional Chinese Medicine, Shantou, 515000 Guangdong China

**Keywords:** Oesophageal cancer, Surgical oncology

## Abstract

To investigate the prognostic value of occult lymph node metastases (OLNMs) in patients with pathologically lymph node negative (pN0) esophageal squamous cell carcinoma (ESCC). OLNMs were detected in 516 pN0 ESCC patients by immunohistochemical staining. The correlation between the clinicopathological features and OLNM, and the prognostic value of OLNM was explored. Eighty-eight patients (17.1%) had OLNMs, including 37 patients with isolated tumor cells (ITCs), 49 patients with micrometastases, and 2 patients with macrometastases (> 2 mm). The OLNM-positive group had poorer differentiation and a more advanced pT category. The 5-year overall survival and disease-free survival for patients with OLNMs were significantly worse than those of IHC-negative patients (P < 0.001), but similar to those of the pN1 patients (P > 0.05). The multivariate analysis showed that OLNM was an independent prognostic factor. In subgroup analyses, the IHC-negative patients had significant survival advantages compared with the ITC group and the micrometastasis group, whereas the survival for the ITC group was similar to that of the micrometastasis group. IHC staining in pN0 ESCC patients might help to identify patients at high risk of death after resection, and ITCs in the lymph nodes appear to have a prognostic value equal to that of micrometastases.

## Introduction

Esophageal carcinoma is one of the leading causes of cancer-related death worldwide^1^. Esophagectomy with appropriate lymphadenectomy remains the major component of therapy for resectable cases. Lymph node status is one of the most important predictors of survival for these patients after curative resection. However, many patients with pathologically lymph node negative (pN0) disease by routine histopathological analysis subsequently develop local recurrence, indicating that undetectable metastatic disease must be initially present in the lymph node at the time of treatment^2^. Thus, detecting these occult lymph node metastases (OLNMs) may improve the staging accuracy so that postoperative treatment may be offered to high-risk patients to decrease the likelihood of recurrent disease.

The Standard assessment procedure for lymph nodes is to cut the nodes serially into 3- to 4-mm thick slices; then hematoxylin and eosin (HE) staining is performed on one to two slices of each lymph node. However, the possibility of missing a small micrometastatic cell cluster because of sampling error or camouflage by normal cells existed^3^. Since the 1980s, investigators have evaluated immunohistochemical (IHC) for cytokeratin and other antibodies to detect these occult metastatic diseases that could not be found by routine HE staining in esophageal carcinoma and many other malignant tumors^4–10^. According to the sixth edition of the American Joint Committee on Cancer (AJCC) staging system, metastatic tumor tissues > 2.0 mm in size are defined as macrometastases, metastatic tumor tissues > 0.2 mm and ≤ 2.0 mm in size are defined as micrometastases, and single cells or groups of tumor cells ≤ 0.2 mm in diameter are defined as isolated tumor cells (ITCs)^11^. Additionally, the definition of ITCs was extended to include the presence of less than 200 cells in a single histologic cross section in the seventh edition of the AJCC staging system^12^. Previous studies have shown that occult metastatic diseases were found in 11.5–61.7% of esophageal carcinoma patients with negative lymph nodes as measured by routine histopathological analysis^2,10,13–19^. However, the patient number in these studies was too small, and most of their patients had adenocarcinomas. Moreover, the study designs and methods of detection were different for most of the previous studies. There is still no consensus on the clinical relevance of these occult metastatic diseases in the lymph nodes of esophageal carcinoma patients.

In this study, we used IHC to detect OLNMs in 516 patients with completely resected pN0 esophageal squamous cell carcinoma (ESCC) and aimed to investigate a possible correlation between these occult metastatic diseases and the prognosis of ESCC patients.

## Patients and methods

### Patients

**Figure 1:**
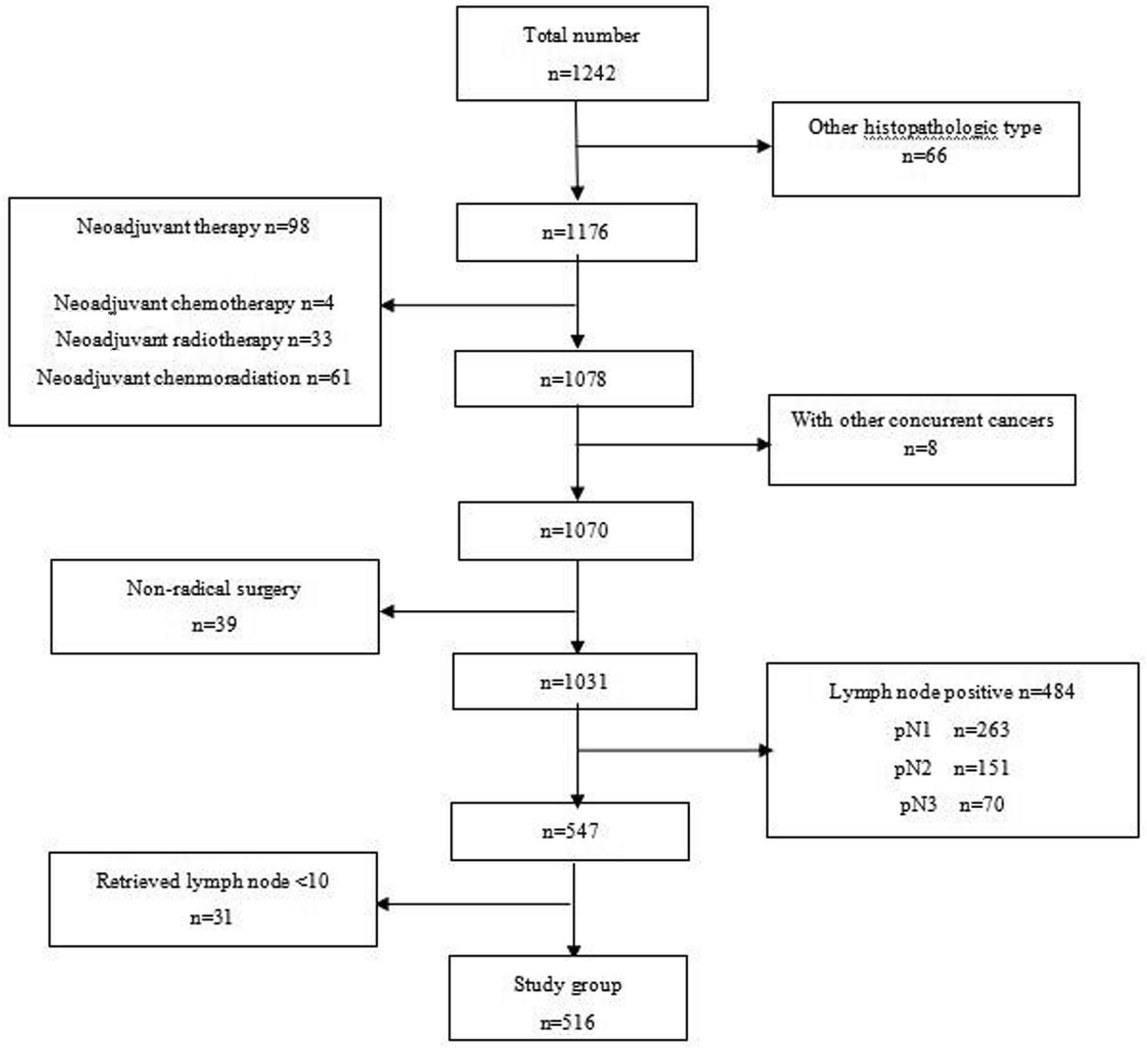
Patients with esophageal cancer underwent surgical resection in the Cancer Hospital of Shantou University Medical College between July 2004 and June 2014.

This study was approved by the Ethics Committee of the Cancer Hospital of Shantou University Medical College (CH-SUMC). All methods were performed in accordance with the approved guideline. All participants signed an informed consent form before they entered this study. Between July 2004 and June 2014, a total of 1242 patients with esophageal cancer were admitted to a single treatment team, supervised by Dr. Yu-ping Chen in the thoracic surgery department of CH-SUMC, and received surgery. We excluded 66 patients with a histopathological diagnosis of non-ESCC, 98 patients who received neoadjuvant therapy, 8 patients with other concurrent cancers, and 39 patients who underwent nonradical surgery, leaving a study group of 1031 patients. Of these, 547 patients were classified as pathologically lymph node negative (pN0) by routine histopathological analysis, 263 patients were classified as pN1, 151 patients were classified as pN2, and 70 patients were classified as pN3 (Fig. 1). We further excluded 31 cases with pN0 disease whose retrieved lymph node number were less than 10, leaving a study group of 516 patients.

### Surgery

Esophagectomy with lymphadenectomy was performed via a left thoracotomy for most patients before 2010, while a right thoracotomy was routinely performed for all patients after 2011; thoracoscopic esophagectomy was also performed after 2011. A standard abdominal lymphadenectomy (left and right paracardial regions, along the lesser curve and the left gastric artery) and mediastinal lymphadenectomy (subcarinal, para-esophageal and thoracic duct, lower posterior mediastinum, pulmonary ligament) was performed in all patients. The lymph nodes around the left and right recurrent laryngeal nerve were also undertaken in patients received a right thoracotomy. Cervical lymphadenectomy was not systematically undertaken.

### Histopathology

**Figure 2:**
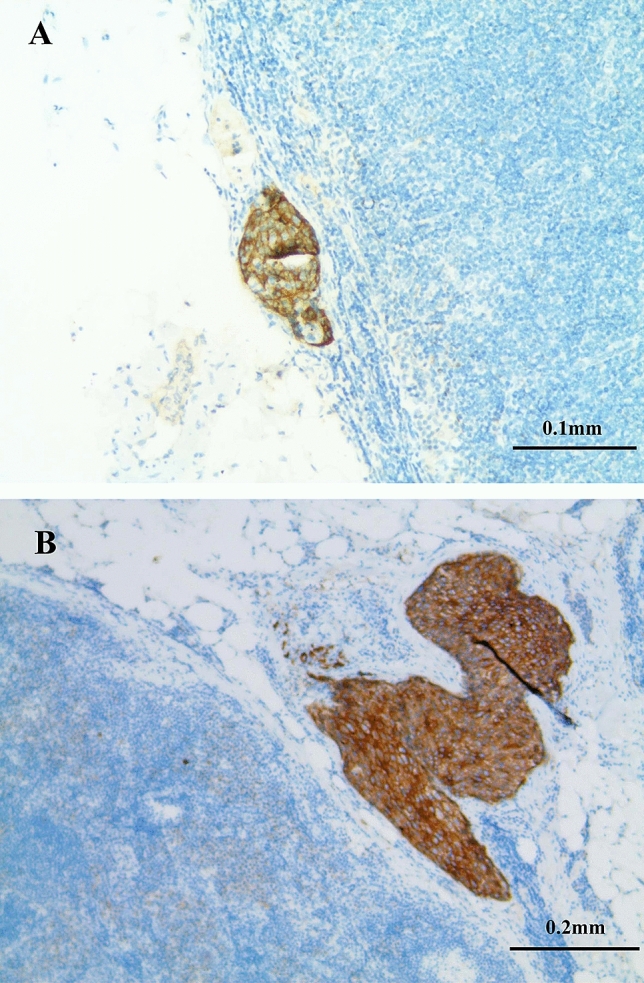
(**A**) Isolated tumor cells in lymph nodes, detected by immunohistochemical for AE1/AE3 (the size ≤ 0.2 mm). (**B**) Micrometastases in lymph nodes, detected by immunohistochemical for AE1/AE3 (the size > 0.2 mm and ≤ 2.0 mm).

The resected lymph node specimens were examined with HE staining and IHC. IHC was conducted according to standard histological protocols in our hospital which had been described previously^20^. All specimens were checked by two experienced pathologists. Two levels at a distance of 250 μm were cut from each lymph node, and two 4 μm thick sections at each level were taken. The 1st and 3rd sections were HE-stained, and the 2nd and 4th sections were stained for IHC with the anti-epithelial-cell antibody AE1/AE3 (MXB, Fuzhou, China). To confirm the sensitivity of IHC, we used the normal esophageal mucosa, which was positive staining for AE1/AE3. The positive finding of OLNMs was noted by the appearance of brownish colored foci in the lymph nodes after IHC staining (Fig. 2). Metastatic tumor tissues > 2.0 mm in size were defined as macrometastases, metastatic tumor tissues > 0.2 mm and ≤ 2.0 mm in size were defined as micrometastases, and single cells or groups of tumor cells ≤ 0.2 mm in diameter or a cluster of less than 200 cancer cells in a single histologic cross section were defined as ITCs.

### Statistical analysis

Lymph node metastases that were greater than 2 mm in IHC stained sections were reclassified as overt lymph node metastases, while ITCs and micrometastases were defined as occult lymph node metastases. SPSS 16.0 software (SPSS Inc., Chicago, IL, USA) was used for the statistical analysis. Overall survival (OS) was calculated from the date of surgery to the date of death or most recent follow-up by the Kaplan–Meier method. Disease-free survival (DFS) was defined as survival without recurrent or metastatic diseases. Differences in survival between groups were calculated by the log-rank test. The multivariate analysis was performed using Cox regression with the factors that were significant on univariate analysis. All tests were two-sided, and a P-value of < 0.05 was considered significant.

## Results

### Patient characteristics

**Figure 3:**
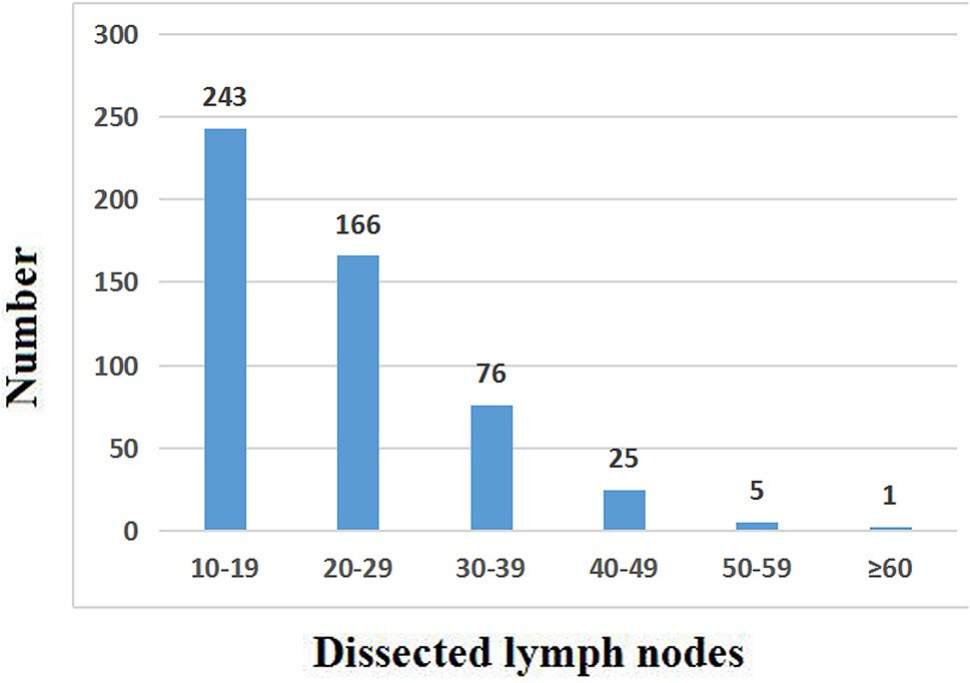
The distribution of the number of lymph node.

The clinicopathological features of 516 pN0 patients are shown in Table 1. There were 366 men and 150 women with a median age of 58 years (range 36–78 years). According to the eighth edition of the AJCC staging system for ESCC, the study group included 80 pT1 patients, 120 pT2 patients, 269 pT3 patients, and 47 pT4 patients. Three hundred and forty-five patients underwent esophagectomy via a left thoracotomy, while the other 171 patients underwent a right thoracotomy or thoracoscopic esophagectomy. A total of 11,578 lymph nodes were retrieved with a median number of 20 (range 10–69) per patient, and the distribution of the number of lymph nodes was shown in Fig. 3. Two patients (0.4%) died within 30 days after surgery. None of these patients received adjuvant chemotherapy after the surgery.

**Table 1:** Correlation between the clinicopathological features and occult lymph node metastases (n = 516).

	No. patients	OLNM		*P* value
		Positive (cases, %)	Negative (cases, %)	
**Gender**				0.606
Male	366	65 (17.8%)	301 (82.2%)	
Female	150	23 (15.3%)	127 (84.7%)	
**Age ** (**year)**				0.276
≤ 60	323	60 (18.6%)	263 (81.4%)	
> 60	193	28 (14.5%)	165 (85.5%)	
**Tumor location**				0.211
Upper third	77	10 (13.0%)	67 (87.0%)	
Middle third	371	70 (18.9%)	301 (81.1%)	
Lower third	68	8 (11.8%)	60 (88.0%)	
**Tumor length**				0.198
≤ 5 cm	365	57 (15.6%)	308 (84.4%)	
> 5 cm	151	31 (20.5%)	120 (79.5%)	
**Histologic grade**				
Well	205	24 (11.7%)	181 (88.3%)	0.031
Moderate	265	54 (20.4%)	211 (79.6%)	
Poor	46	10 (21.7%)	36 (78.3%)	
**Operation approach**				0.534
Left thoracotomy	345	56 (16.2%)	289 (83.8%)	
Right thoracotomy	171	32 (18.7%)	139 (81.3%)	
**pT category**				0.024
pT1	80	5 (6.3%)	75 (93.7%)	
pT2	120	19 (15.8%)	101 (84.2%)	
pT3	269	53 (19.7%)	216 (80.3%)	
pT4	47	11 (23.4%)	36 (76.6%)	

*OLNM* occult lymph node metastases.

### IHC examination

A total of 88 patients (17.1%) were found to have IHC-positive lymph nodes, including 37 (7.2%) who had ITCs, 49 (9.5%) who had micrometastases, and 2 (0.4%) who had macrometastases. Of the 11,578 “tumor negative” lymph nodes, 94 (0.8%) were IHC-positive (82 patients with one positive lymph node and 6 patients with two positive lymph nodes). A larger tumor deposit was recorded when patients had more than one positive lymph node. Of these 94 positive lymph nodes, 80 (85.1%) had tumor deposit on the first section, while the other 14 (14.9%) only found in the second section. OLNMs were found to be associated with histologic grade and pT category. The positive group had poorer differentiation and a more advanced pT category. However, gender, age, tumor length, operation approach, and tumor location were not associated with occult disease (Table 1).

### Survival and prognostic factors

Follow-up continued until January 2019. The mean follow-up time was 69.9 months (range 1–174 months). One hundred and eighty-seven patients died during the follow-up, 315 were still alive, and 14 were lost to follow-up (2.7%).

The 5-year OS rate and 5-year DFS rate were 69.0% and 66.5%, respectively, for all of the 516 patients. The variables related to OS and DFS are shown in Table 2. The 5-year OS and DFS for patients with OLNMs were 53.9% and 50.5%, respectively, compared with those of the IHC-negative patients (72.1% and 69.9%; P < 0.001). Gender, age, tumor location, tumor length, histologic grade, and pT category were other factors that affected DFS (P < 0.05), while tumor location did not affect OS (P > 0.05). The 5-year OS and DFS for patients with ITCs or micrometastases (excluding 2 patients with macrometastases) were 55.2% and 51.7%, respectively, which were also significantly worse than the rates in patients with negative lymph nodes (P < 0.001).

**Table 2:** Univariate analysis in regard to overall survival and disease-free survival according to patient and tumor characteristics.

Variable	No. patients	MST (months)	5-year OS (%)	*P* value	5-year DFS	*P* value
**Gender**				0.002		0.038
Male	366	127	66.0		64.5	
Female	150	–	76.2		71.6	
**Age (year)**				0.001		0.024
≤ 60	323	–	74.0		70.1	
> 60	193	127	60.5		60.5	
**Tumor location**				0.054		0.041
Upper third	77	118	57.4		56.6	
Middle third	371	–	69.3		66.5	
Lower third	68	144	79.3		77.8	
**Tumor length**				0.020		0.001
≤ 5 cm	365	–	71.0		69.9	
> 5 cm	151	115	64.1		58.5	
**Histologic grade**				< 0.001		< 0.001
Well	205	–	78.0		76.6	
Moderate	265	118.0	64.0		60.8	
Poor	46	74.0	56.9		54.5	
**Operation approach**				0.062		0.075
Left thoracotomy	345	135.0	67.6		65.1	
Right thoracotomy	171	–	71.9		69.3	
**OLNM**				< 0.001		< 0.001
Negative	427	–	72.1		69.9	
Positive	89	67.0	53.9		50.5	
**pT category**				< 0.001		< 0.001
pT1	80	–	89.8		89.9	
pT2	120	140.0	69.4		69.6	
pT3	269	146.0	66.5		64.4	
pT4	47	52.0	45.3		31.9	

*DFS* disease-free survival, *MST* median survival time, *OS* overall survival, *OLNM* occult lymph node metastases.

The multivariate analysis revealed that OLNM was an independent factor for OS (Hazards ratio [HR] 1.805, P < 0.001, Table 3) and DFS (HR 1.722, P = 0.001, Table 3). The OLNM-positive group had significantly shorter OS and DFS. Other independent factors for OS and DFS were gender, age, histologic grade, and pT category; tumor location also affected DFS.

**Table 3:** Multivariate Cox regression analysis in regard to overall survival and disease-free survival of the 516 patients with pN0 esophageal squamous cell carcinoma.

Prognostic factor	Hazards ratio	95% CI	*P* value
**Overall survival**			
Gender	0.521	0.363–0.745	< 0.001
Age	1.681	1.257–2.249	< 0.001
Tumor length	1.137	0.829–1.561	0.425
Histologic grade	1.633	1.297–2.056	< 0.001
pT category	1.507	1.235–1.840	< 0.001
OLNM	1.805	1.299–2.506	< 0.001
**Disease-free survival**			
Gender	0.640	0.463–0.885	0.007
Age	1.492	1.123–1.981	0.006
Tumor location	0.665	0.503–0.880	0.004
Tumor length	1.318	0.975–1.782	0.073
Histologic grade	1.619	1.297–2.020	< 0.001
pT category	1.712	1.409–2.081	< 0.001
OLNM	1.722	1.260–2.354	0.001

*CI* confidence interval.

**Figure 4:**
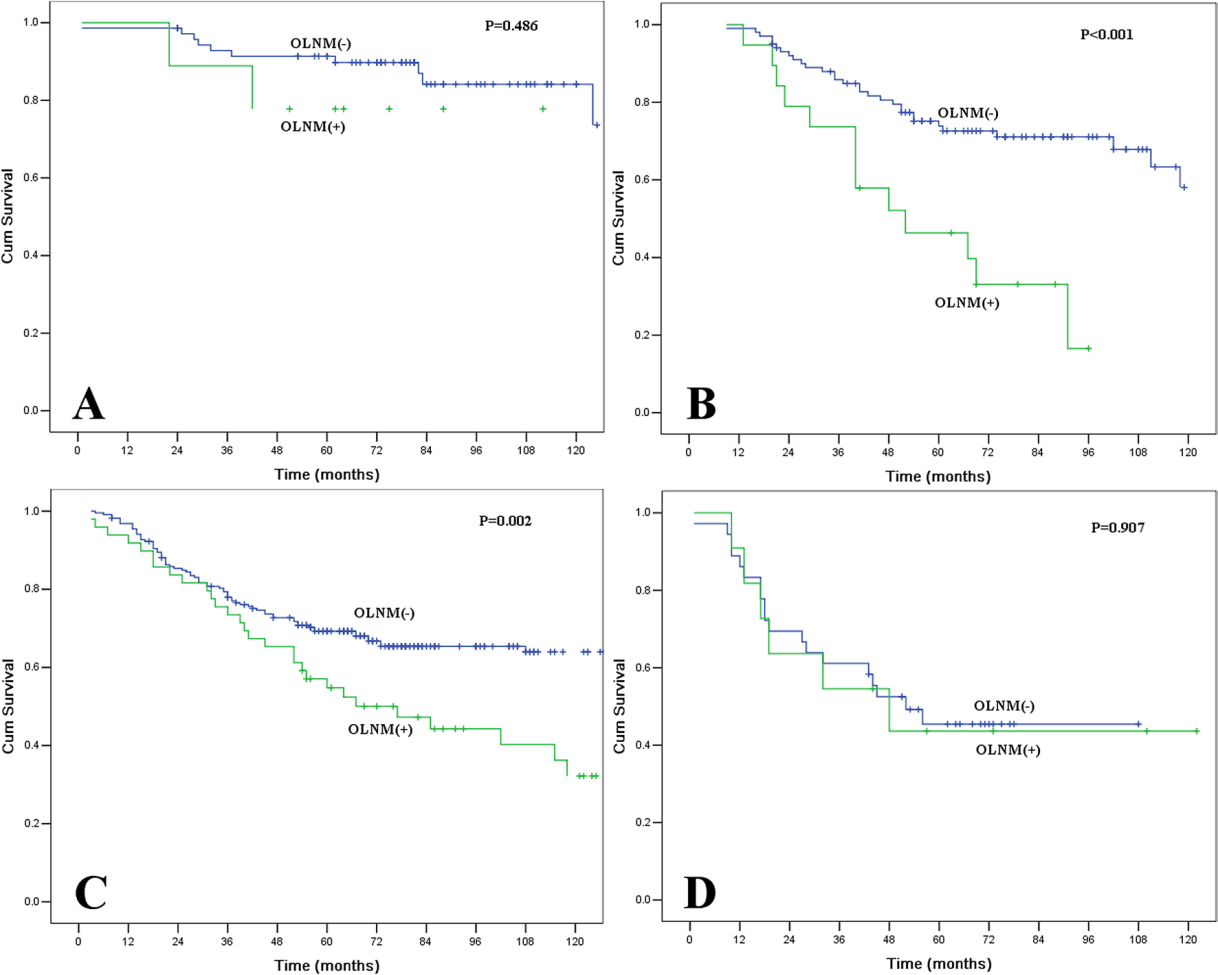
(**A**) The impact of occult lymph node metastasis (OLNM) on patients with pT1 category. The difference in overall survival was not significant (P = 0.486). (**B**) The impact of OLNM on patients with pT2 category. The difference in overall survival was significant (P < 0.001). (**C**) The impact of OLNM on patients with pT3 category. The difference in overall survival was significant (P = 0.002). (**D**) The impact of OLNM on patients with pT4 category. The difference in overall survival was not significant (P = 0.907).

Moreover, we compared the impact of OLNM on patients with different pT categories (Fig. 4). The OLNM-positive group had significantly shorter OS in patients with pT2 diseases (P < 0.001) and pT3 diseases (P = 0.002), while no survival differences were observed between OLNM-negative and OLNM-positive groups in patients with pT1 diseases (P = 0.486) and pT4 diseases (P = 0.907). However, the number of patients with pT1 diseases (80 patients) and pT4 diseases (47 patients) was relatively smaller than the other two groups, and only 5 patients with pT1 category and 11 patients with pT4 category had positive OLNM.

**Figure 5:**
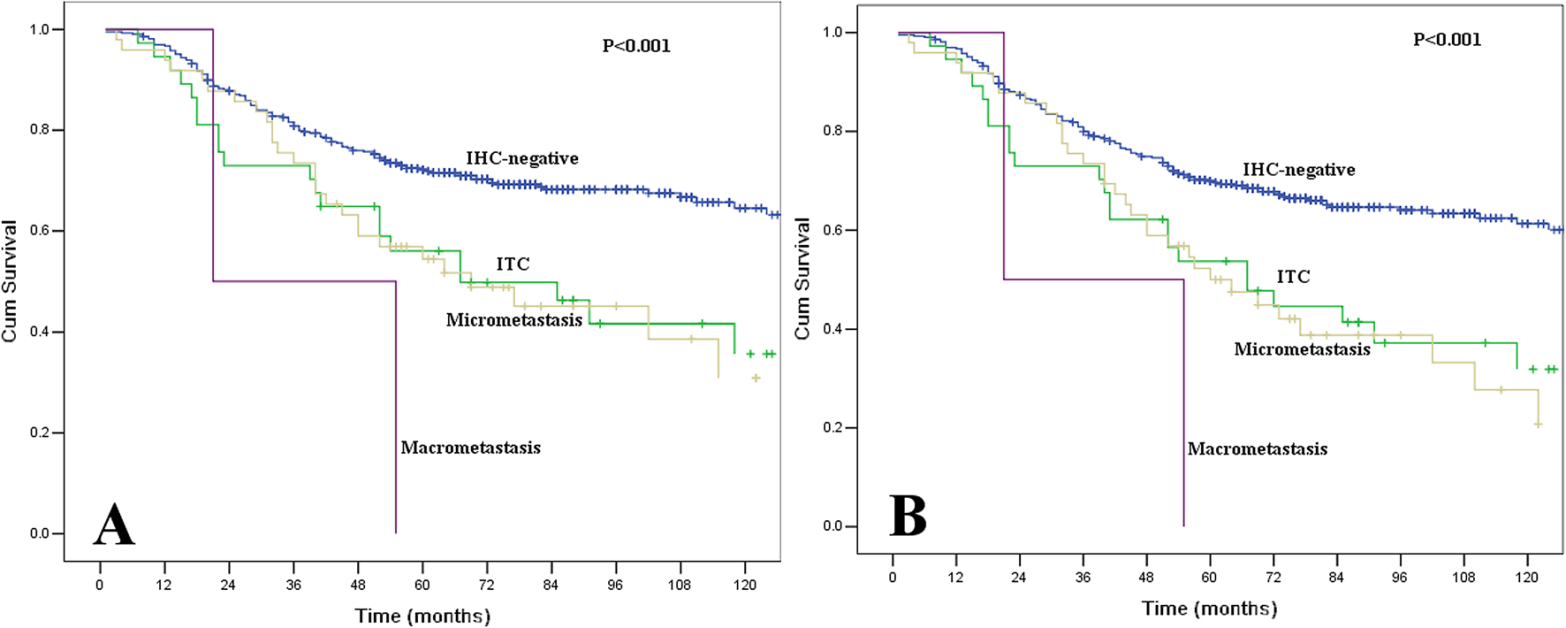
(**A**) Comparisons of overall survival between different tumor deposit groups. The survival was significantly different across these four groups (P < 0.001). (**B**) Comparisons of disease-free survival between different tumor deposit groups. The survival was significantly different across these four groups (P < 0.001).

Furthermore, we compared survival between different tumor deposit groups. The OS and DFS were significantly different across these four groups (P < 0.001, Fig. 5). In subgroup analyses, the IHC-negative patients had significant survival advantages compared with the other three subgroups of IHC-positive patients (Table 4). However, no significant survival differences were found between the three subgroups of IHC-positvie patients (P > 0.05).

**Table 4:** Subgroup survival analyses between different tumor deposit group in lymph nodes.

Strata	Comparison	χ^2^	*P* value
IHC-negative	ITC	10.467	0.001
IHC-negative	Micrometastasis	10.242	0.001
IHC-negative	Macrometastasis	5.763	0.017
ITC	Micrometastasis	0.001	0.981
ITC	Macrometastasis	1.676	0.195
Micrometastasis	Macrometastasis	2.241	0.134

**Figure 6:**
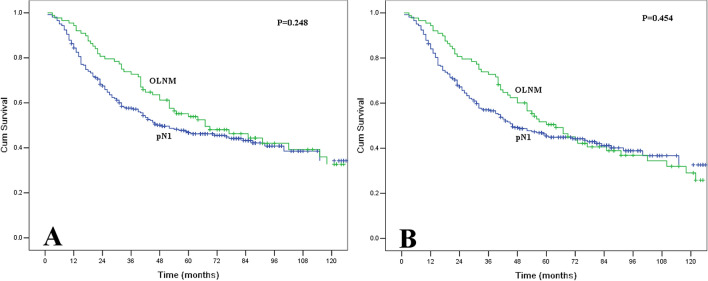
(**A**) Comparisons of overall survival between the patients with occult lymph node metastases and patients with pN1 by routine histopathological analysis. The differences in survival were not significant (P = 0.248). (**B**) Comparisons of disease-free survival between the patients with occult lymph node metastases and patients with pN1 by routine histopathological analysis. The differences in survival were not significant (P = 0.454).

Finally, we assessed the difference in prognosis between the patients with OLNMs and the 263 patients with pN1 by routine histopathological analysis. The pN1 group contained 198 men and 65 women with a median age of 57 years (range 35–78 years). According to the eighth edition of the AJCC staging system for ESCC, the pN1 group included 10 pT1 patients, 33 pT2 patients, 181 pT3 patients, and 39 pT4 patients. The 5-year OS and DFS for pN1 patients were 46.8% and 45.3%, respectively. The patients with OLNM had no significant differences in OS (P = 0.248) or DFS (P = 0.454) compared with the pN1 patients (Fig. 6). Furthermore, we also compared the survival of pN1 patients between the different OLNM tumor deposit groups. However, no significant differences in OS (P = 0.434) and DFS (P = 0.624) were found between these four groups.

## Discussion

Although ITCs and micrometastases in lymph nodes were included in the current AJCC staging system for breast cancer, they were not designated as staging parameters in esophageal cancer. While some previous studies have found that occult diseases in the lymph nodes are associated with poor outcomes in patients with esophageal cancer^2,10,13–16^, others have not^17–19^. However, the number of patients in these previous studies was too small. Moreover, most of these studies did not discriminate between ITCs and micrometastases. We think that more evidence is needed to confirm the impact of these occult diseases on esophageal cancer patients to make relevant treatment decisions.

To the best of our knowledge, we reported the largest-ever patient cohort to investigate the prognostic significance of OLNM in esophageal cancer detected by IHC staining. Notably, all of the patients enrolled in our study had ESCC and underwent radical resection. We also used a standardized method to distinguish these occult metastasis diseases as ITCs (diameter ≤ 0.2 mm) or micrometastases (diameter > 0.2 mm and ≤ 2.0 mm). The occult metastatic disease rate of 17.1% in our study was within the range reported in previous studies (11.5–61.7%)^2,10,13–19^. We also found that OLNM was associated with histologic grade (P = 0.031) and pT category (P = 0.024), but was not associated with gender, age, tumor location, or tumor length. It is easy to understand that lymph node metastasis is correlated with histologic grade and the depth of tumor invasion in esophageal cancer patients, but whether this correlation existed in the OLNMs is controversial. Although some of the previous studies showed a trend towards a higher frequency of OLNMs in patients with higher pT stage or poorer differentiated diseases^14,21^, the majority of studies did not demonstrate any correlation between histologic grade or pT stage and the prevalence of OLNM^10,17,18,22^. However, as all of these previous studies enrolled a relatively small number of patients, we think that they might not have been able to detect the possible correlation between OLNMs and different clinicopathological features.

In our study, the patients with OLNMs were found to have significantly worse survival than those who remained node negative after IHC staining, which was similar to reports from previous studies^2,10,13–16^. Although the 5-year OS and DFS of 53.9% and 50.5%, respectively, for patients with OLNM were higher than the rates for pN1 patients (46.8% and 45.3%, respectively), the differences were not significant. Our results indicated that IHC staining in ESCC patients with negative lymph nodes might help to identify patients at high risk of death after resection, and all of these IHC-positive staining diseases should be categorized as pN1, rather than pN0. The detection of OLNMs in patients with pN0 ESCC might be a useful adjunct to the current TNM stage for ESCC.

Micrometastases in lymph nodes have been reported to be correlated with poor outcomes in patients with breast cancer and other malignant tumors^3,4,7,23^, however, the influence of ITCs on prognosis is more controversial. Some studies have shown that ITCs had no histologic evidence of malignant activity and were not a predictor of overall survival^24–26^. In the seventh and eighth edition of the AJCC staging system for breast cancer, T1N0(i +)M0 disease was equivalent to T1N0M0 disease, and both were categorized as stage IA, while T1N1miM0 disease was categorized as stage IB, indicating that the ITCs in the lymph node did not influence survival for T1 breast cancer patients. However, few studies have reported survival differences between patients with ITCs and micrometastases in esophageal cancer. One study by Thompson et al.^2,1^ found that patients with ITCs had survival rates similar to patients with micrometastases in esophageal cancer, and both groups had a worse prognosis than patients with negative lymph nodes. However, most of the patients (74%) had esophageal adenocarcinoma, and the authors did not conduct a subset analysis for patients with ESCC. Moreover, 59% of the patients in their study underwent neoadjuvant treatment, and the median number of lymph nodes per patient was only 5. As neoadjuvant therapy might reduce the tumor size (including the metastatic lymph node) or OLNMs, some of these occult metastases in lymph nodes after neoadjuvant therapy might once been overtly metastatic. The relatively limited lymphadenectomy might have also understaged the patients and undermined the conclusions made by the authors.

In the current study, we also found no significant survival differences between patients with lymph node ITCs and patients with micrometastases. Moreover, after comparing the survival of these patients with the pN1 patients, we did not find significant differences. It seems that it is not important to distinguish ITCs and micrometastases in ESCC patients. One possible reason to explain the association between ITCs and survival observed in our study was that the ESCCs were very aggressive and did not require long follow-up times to observe the survival differences for these patients^21,27,28^.

Our study has some limitations. First, it was a single-center, retrospective study. There is still no consensus on how to determine the presence of OLNMs. It is difficult to compare the results between studies because of the substantial variation in study designs and detection techniques. To analyze and compare results of different studies, it is crucial to standardize the definitions and methods used. Second, although IHC is relatively simple, issues were raised in determining how many sections were sufficient for the detection of OLNMs. We chose two levels for each lymph node in our current study, as Turner et al.^2,9^ recommended in breast cancer. A previous study showed that 29% of patients had occult nodal metastases detected on the second or third section only, indicating that nearly one-third of patients with lymph node metastatic diseases were missed by detection methods using only one single section^2,1^. On the other hand, Märkl et al.^9^ found that 16% of patients with micrometastases in the lymph node were macrometastases larger than 2 mm after three additional step sections (distance, 200 μm) were cut. In our current study, of the 94 positive lymph nodes, 80 (85.1%) had tumor deposit on the first section, while the other 14 (14.9%) only found in the second section. We think that serial sections may increase the amount of nodal tissue examined and consequently minimize the false negative or positive rate.

In conclusion, IHC staining in ESCC patients with negative lymph nodes might help to identify patients at high risk of death after resection. The patients with OLNMs had significantly poorer survival than those who remained node negative after IHC staining but similar survival to the pN1 patients. ITCs in the lymph nodes appear to have a prognostic value equal to that of micrometastases in patients with pN0 ESCC. Further prospective studies are required to examine our findings and evaluate the value of adjuvant therapy for ESCC patients with OLNMs.
